# Goal Pursuit in Youth with Chronic Pain

**DOI:** 10.3390/children3040036

**Published:** 2016-11-22

**Authors:** Emma Fisher, Tonya M. Palermo

**Affiliations:** 1 Center for Child Health, Behavior, and Development, Seattle Children’s Research Institute, Seattle, WA 98122, USA; Tonya.Palermo@seattlechildrens.org; 2 Department of Anesthesiology and Pain Medicine, University of Washington, Seattle, WA 98195, USA

**Keywords:** adolescents, children, chronic pain, goal pursuit

## Abstract

Children and adolescents frequently experience chronic pain that can disrupt their usual activities and lead to poor physical and emotional functioning. The fear avoidance model of pain with an emphasis on the maladaptive behaviors that lead to activity avoidance has guided research and clinical practice. However, this model does not take into consideration variability in responses to pain, in particular the active pursuit of goals despite pain. This review aims to introduce a novel conceptualization of children’s activity engagement versus avoidance using the framework of goal pursuit. We propose a new model of Goal Pursuit in Pediatric Chronic Pain, which proposes that the child’s experience of pain is modified by child factors (e.g., goal salience, motivation/energy, pain-related anxiety/fear, and self-efficacy) and parent factors (e.g., parent expectations for pain, protectiveness behaviors, and parent anxiety), which lead to specific goal pursuit behaviors. Goal pursuit is framed as engagement or avoidance of valued goals when in pain. Next, we recommend that research in youth with chronic pain should be reframed to account for the pursuit of valued goals within the context of pain and suggest directions for future research.

## 1. Introduction and Purpose of Review

Children and adolescents frequently experience pain, most commonly headache, abdominal pain, and musculoskeletal pain [1]. Pain can range in intensity, frequency, and duration. For some children and adolescents, pain can persist, interrupting daily routines and activities. Pain that persists for longer than three months is defined as chronic [2]. Between 5%–8% of children experience chronic pain that is severe and disabling [3]. Chronic pain during childhood is a risk factor for chronic pain in adulthood, with studies showing that 35% of adolescents with chronic pain go on to report chronic pain in adulthood [4] and experience emotional distress [5]. Thus, understanding and treating pain in childhood is critical for interrupting a potential lifelong trajectory of pain and disability.

Children and adolescents with chronic pain report that pain is disruptive to many aspects of their daily functioning, such as decreasing physical functioning, impacting school attendance, and interactions with peers, leading to emotional distress [6,7,8]. In particular, children and adolescents with chronic pain report high levels of anxiety, depression, and maladaptive coping strategies [9,10,11,12]. Greater anxiety symptoms have been linked to avoidance of activities [13]. Unlike acute pain where children are encouraged to rest, or avoid activities that may aggravate the injury, treatment of chronic pain requires encouraging children to engage with physical activities and normal functioning despite their pain. Avoidance of everyday activities leads to increased disability, increased depression, higher fear, and increased pain sensitivity [7,13,14,15].

The fear avoidance model demonstrates this pathway linking anxiety and avoidance, and was proposed to aid research and clinical understanding of the maintenance of pain and disability [14]. In a second pathway, confrontation is predicted to lead to recovery; however, this has received less research attention. This model has been more recently adapted to describe development and mechanisms of pain in children and adolescents [13]. However, there are some limitations to this model, particularly in the underlying assumption that avoidance is a single pathway that leads to disability. Children and adolescents may or may not avoid activities because of pain, and there is evidence to suggest that many children and adolescents persist with activities despite their pain. As documented in epidemiological papers, around 25%–40% of children report experiencing chronic pain [3,16]. However, only 5%–8% of children have moderate to severe chronic pain, which impacts their daily functioning [3]. Therefore, many children and adolescents have frequent pain that is not disabling, and there may be unique protective factors that lead to recovery or adaptation to pain. The fear avoidance model does not conceptualize the experiences of children and adolescents with chronic pain who demonstrate more adaptive functioning or who are not fearful. This gap in understanding of adaptation to pain may be critical to expanding and informing psychological treatment models for children and adolescents with chronic pain.

Recognizing this variability in children’s responses to chronic pain, we propose a new conceptual model that characterizes an individual’s reaction to pain in the context of their engagement and pursuit of personal goals. We propose to reframe our thinking about young people with chronic pain to specifically understand those factors that lead to engagement and avoidance, which better reflects the active and dynamic process of responding to challenges in the pursuit of personal goals. Specifically, we focus on the interplay of child individual processes (such as the child’s motivation and fear of pain) and parent factors (such as parent expectations and anxiety) in the pursuit of goal achievement that results in approach or avoidance of activities. In this review, we describe the work to date that has investigated goal pursuit, importance, and frustration within the context of pediatric chronic pain/illness. Next, we propose a new conceptual model that integrates this research. Finally, we suggest next steps for future research on this topic.

## 2. An Overview of Goals, Goal Achievement, and Goal Frustration

Goals, defined by Austin and Vancouver [17], are “internal representations of desired states, where states are broadly construed as outcomes, events, or processes” (p. 338). Goals can be short or long-term and can pertain to any area such as academics, social, and sporting. Some goals may transcend health and illness; however, chronic pain may lead to a re-prioritization of goals, the creation of new goals, or the termination of existing goals.

When pain is experienced, some goals may become unattainable. For example, a painful limb due to a fracture may prevent a child from taking part in a sports tournament. When pain persists, the consequences may include a lack of attainment of the child’s short-term and long-term goals. For example, pain may prevent regular attendance at school, impacting later educational attainment. Research in adults has suggested that giving up unattainable goals can be positive for mental health outcomes [18]; however, it is unknown when it is appropriate or desirable for children to “give up” their goals, even if deemed unattainable or difficult to attain. Nevertheless, the conflict between having pain and wanting to engage in desired activities is not well understood in this field and is central to developing effective treatment strategies to motivate young people to pursue goal-directed activities despite their pain.

Experiencing pain for a long period of time can lead to goal frustration, regardless of goal importance. Two studies have investigated goal frustration in youth with chronic pain, one including youth with and without headaches [19] and one including youth with and without musculoskeletal pain [20]. In both studies, youth rated goal importance and frustration on six categories including personal values, social acceptance, self-acceptance, school, health, and self-development. In a study by Massey, Garnefski and Gebhardt [19], youth with weekly headaches allocated a higher importance to personal goals (e.g., treating others fairly and having a good relationship with parents) compared with those without headaches. Goal importance did not differ for other categories (e.g., social acceptance, self-acceptance, school, and health). Adolescents with weekly headaches reported higher frustration on social- and self-acceptance, school, and health goals compared with adolescents without headaches. However, adolescents with weekly headaches did not report higher frustration for personal goals compared to those without headaches.

Stommen, Verbunt, and Goossens [20] similarly found no significant differences in how adolescents allocated the importance of goals between those with and without musculoskeletal pain. However, adolescents with musculoskeletal pain reported higher frustration with goals pertaining to personal values, social acceptance, self-acceptance, and health compared with adolescents without musculoskeletal pain. In both studies, increased goal frustration was associated with higher depression and lower quality of life [19,20]. These findings suggest that, while there are few differences in adolescent perceptions of goal importance, there seems to be a relationship between higher goal frustration and poorer well-being. However, it is unknown whether frustration is associated with a lower pursuit of goals, or whether it encourages adolescents to strive more persistently towards their goals. More likely, other individual differences will predict the pursuit or avoidance of these goals.

Goals have a specific context that is important to understand. There are often multiple, competing, and conflicting goals that must be weighed by the individual in determining their action toward those goals. In a study with 170 adolescents from a community sample, Fisher et al. [21] investigated conflicting goals. Adolescents were asked to report their likelihood of approaching or avoiding situations where pain conflicted with a goal (e.g., having a headache but wanting to meet up with friends). Vignettes described either high (the headache is very painful) or low (the headache is quite mild) pain intensity situations. Afterwards, adolescents were asked to report the personal importance they ascribed to each goal described in the vignettes. Results showed that activities with a higher goal importance were more likely to be approached. However, individuals who had higher levels of pain anxiety were more likely to avoid high pain intensity situations even if the goal was very important to them. General anxiety was also assessed, but this was not a significant predictor of avoidance, suggesting that pain-specific anxiety may more directly influence goal engagement.

Although adolescence is a time of increasing emotional and behavioral autonomy and separateness from parents [22], parents are still important role models and their communication and modeling are critical in the context of chronic pain. Chronic pain has a bi-directional impact on parents, as proposed in the integrated parent and family model. Specifically, this model outlines that the individual child characteristics lie within broader dyadic and family level factors that influence a child’s experience of pain and associated disability [23]. Parents of youth with chronic pain report a higher burden of parenting, higher emotional distress, and lower social functioning when their child has chronic pain [22,24]. However, specific parenting behaviors and modeling also influence a child’s interpretation of pain and pain-related behaviors [25]. Some parent behaviors have been associated with poorer child adaptation to chronic pain, such as communicating too much about pain to their child, catastrophizing about their child’s pain, modeling illness behaviors, or any combination of these. For example, parents who reported higher levels of catastrophizing were more likely to stop their child from doing a task earlier than parents with lower catastrophizing [26]. Moreover, a relationship has been demonstrated between parents’ higher degree of attending behaviors toward their child’s pain and higher levels of child symptom complaints, disability, and depression [25,27].

Parents can be a source of support and motivation when their child is striving for their desired goals. Research in children with diabetes has found that parents who supported their children in their treatment were more likely to reach their blood glucose targets compared with those children whose parents did not [28]. Similarly, Fisher et al. [29] found in a self-management cognitive-behavioral therapy intervention for youth with chronic pain and their parents that dyads who chose matching goals (i.e., identical goal content) at the start of treatment were more likely to report lower pain intensity post-treatment and at follow-up. In particular, dyads with identical physical activity goals were more likely to have lower pain intensity at post-treatment and follow-up were comparable to those dyads who selected less physically active goals or did not agree on goal content.

Within the context of pain, most prior research investigating goals has been conducted within adult populations. These studies found that pain severity and catastrophizing about pain consistently predicted task interference [30] and that fear of pain mediated associations between goal self-efficacy/conflict and depression/disability in adults with chronic low back pain [31]. Other research has proposed theoretical models of problem-solving during goal pursuit [32]. This model proposes that worry is a key psychological factor in problem-solving around chronic pain. 

Further, there have been several adaptations of the fear avoidance model using a motivational perspective to understand adults with chronic pain [32,33,34,35]. An updated fear-avoidance model incorporating the role of goals has been published [36]. This model predicts that pain can be either high or low threat to an individual, who will then choose to engage valued life goals or pain control goals. When priority is given to valued goals, this leads to approach behaviors and then recovery. When priority is given to pain control goals, it is predicted that this leads to the fear, avoidance, and disability pathway of the fear avoidance model [36]. Investigations into valued goals vs. pain control goals have been explored in adults [37,38], but not in children. The model proposed by Vlaeyen, Crombez, and Linton [36] is the first to explicitly incorporate goals within the fear avoidance model; however, it does not account for the developmental context (i.e., parent factors and developmentally relevant child factors) that is important to understanding chronic pain and goal pursuit in children and adolescents. There is also research within the context of chronic illnesses theorizing goal pursuit and health behavior change [39,40]. However, our model presented here differs from other models in the field as we focus specifically on factors that might promote or inhibit goal pursuit when children experience pain, rather than using goal pursuit to understand disability or to describe behavior change, goal setting, or other related process. 

## 3. Description of a New Goal Pursuit Model for Pediatric Chronic Pain

**Figure 1:**
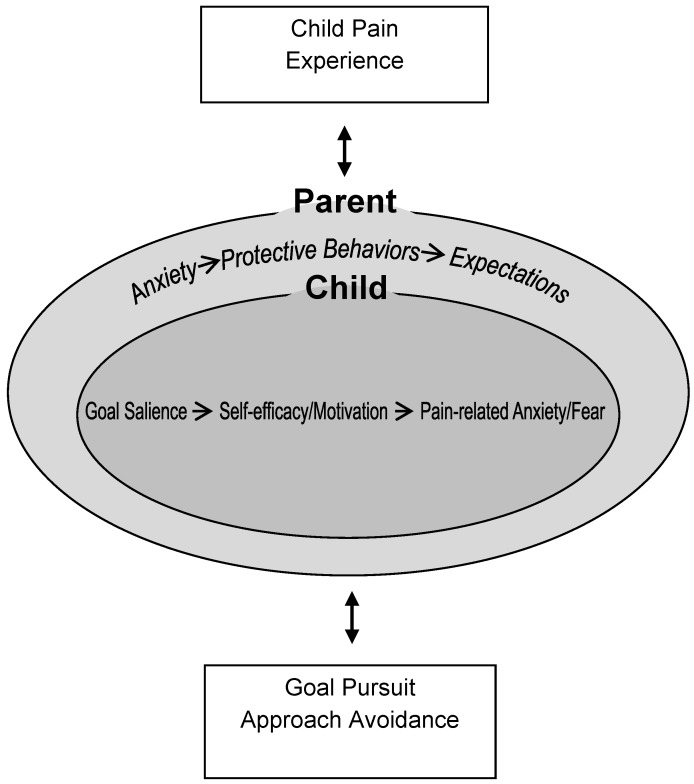
Goal Pursuit Model of Pediatric Chronic Pain.

We propose a new goal pursuit model for pediatric chronic pain (Figure 1), which incorporates the child’s pain experience, child and parent factors, and goal pursuit behaviors. The child’s pain experience is moderated by both child and parent responses to pain, which accumulatively influences goal pursuit behavior. In our proposed model, the child is placed within the broader context of the parent, consistent with a social-environmental framework. Here, we focus on the factors that could inhibit or encourage goal pursuit behavior in youth with chronic pain.

### 3.1. Child’s Pain Experience

The child’s experience of pain is presented as the first step in our model. Definitions of chronic pain in children and adolescents are focused only on the length of time that the child has experienced the pain (i.e., longer than three months [2]). However, many other factors are important to consider about the pain experience such as the child’s perceived intensity/severity of pain, amount of interference, pain frequency, as well as pain duration. The child’s experience of their pain may directly influence whether they engage or avoid a specific goal. Perceptions of more intense or worse pain experiences are more likely to lead to avoidance of goals, compared with less severe pain experiences, as demonstrated in the vignette study conducted by Fisher et al. [41]. However, as shown in Figure 1, the pain experience of the child is also likely to interact with child and parent factors and thus influence goal pursuit behaviors.

### 3.2. Child Factors

Three individual child factors are highlighted in the model including goal salience, self-efficacy/motivation, and pain-related anxiety/fear. Goal saliency, which describes the importance that a child or adolescent allocates to a goal may directly influence motivation toward goal engagement. Goals will differ in salience for each child and may also be context-dependent. Goals with higher salience are more likely to be motivationally pursued and therefore approached compared with those goals that are less salient. However, even if a goal is deemed salient, high pain anxiety or low self-efficacy may influence the child’s pursuit of the goal. Higher levels of pain intensity have also been associated with lower motivation in this population, which may translate into the child avoiding a goal despite having a high level of personal importance (e.g., seeing friends). Of course, goal salience and pain experience may conflict, meaning that, even though a child wants to pursue a goal, pain severity may inhibit goal engagement.

Self-efficacy, or one’s belief in their ability to manage pain, may also be an important individual factor that influences goal pursuit behaviors. Self-efficacy has been studied more frequently in adults with chronic pain, finding consistently that higher self-efficacy is associated with lower levels of disability, distress, and pain severity in a review of 86 studies [42]. Although only a few studies assessing self-efficacy have been conducted in youth with chronic pain, patterns are similar; higher self-efficacy was associated with lower pain intensity [43] and better school functioning, lower disability, and lower depression in youth with chronic headaches [44]. Although there is limited pediatric research on self-efficacy, we predict that higher levels of self-efficacy are more likely to be associated with engagement of goals.

Even if goals are salient and the child has a high level of motivation and self-efficacy, the child’s pain anxiety or fear of pain may inhibit goal pursuit and lead to avoidance behaviors. Since the publication of the fear avoidance model [14], a plethora of research has been conducted to show the association between pain intensity, pain anxiety, fear of pain, and disability. Research has demonstrated that higher levels of pain anxiety and fear of pain increase avoidance behaviors when in pain [13,45,46,47]. In addition, adolescents with higher pain anxiety were likely to avoid activities despite rating goals as important [41]. Pain anxiety/fear of pain may moderate the relationship between goal salience and goal pursuit behaviors.

There are likely many other individual child factors such as neurobiological factors that may also moderate a child’s pain experience and therefore their goal pursuit engagement or avoidance behaviors. However, we chose to focus on several child psychosocial factors that have received research attention and could be specifically targeted in psychological interventions for pediatric chronic pain and disability.

### 3.3. Parent Factors

The influence of parents on the child’s goal pursuit behaviors is critical in a developmentally informed model of childhood chronic pain. Parent factors will influence child factors directly, and any goal pursuit behavior will be a conflict or balance between child and parent factors, and the pain experience.

Parent anxiety, including worry and catastrophizing about their child’s pain has been found to be associated with maladaptive behaviors, such as protectiveness and modeling of illness behaviors [48,49]. Children with chronic pain, whose parents report higher anxiety and are more protective, are also more likely to report higher levels of anxiety and disability [49,50,51]. Similarly, parents who report higher catastrophizing, which is comprised of magnification, rumination, and helplessness about their child’s pain condition, are more likely to stop their child from engaging in activity when they have pain [26]. These parent factors have been associated with higher disability in children reporting chronic pain, and are likely to influence children’s goal pursuit.

Parents with higher anxiety and protective behaviors are likely to reduce their expectations of their child. Parent expectations which are realistic of their child’s abilities are predicted to increase goal approach. Unrealistic expectations such that expectations are set too high or too low are predicted to increase goal avoidance. For example, going to school may be of low salience to the child, but parent expectation may be appropriate and realistic. Such expectations may drive parent behaviors (i.e., reduced protectiveness behaviors) that support the likelihood of the child pursuing the goal. Chronic pain presents in variable ways and often children and those around them have to temporarily shift their expectations of what the child can achieve until pain intensity is manageable. Research to date has predominantly focused on maladaptive parent behaviors (rather than how positive behaviors can support children). For example, research has shown that when parents reduce their expectations considerably, such as excuse children from chores, fail to enforce school, and allow additional rest time, children experience increased pain-related disability [52]. We hypothesize that expectations from others are likely to influence goal salience, with realistic and positive expectations encouraging engagement of goals, while too low or too high expectations likely lead to the avoidance of goals.

### 3.4. Goal Pursuit

When faced with a goal conflict, the individuals’ avoidance or engagement of goals is an observable and measurable behavior. Avoidance can be expressed as the delay of a goal or the termination of the goal such that the goal is not accomplished. Engagement, on the other hand, can be expressed in terms of the extent of involvement in or accomplishment of the goal (i.e., partial or full engagement). After pursuing a goal, the child may experience direct or indirect consequences. For example, when a child or adolescent engages with an activity to achieve a goal, they may experience increased pain intensity as a consequence, which may influence their goal pursuit behavior in the future. Equally, if the child or adolescent engages in goal behavior and experiences rewarding consequences, then they may be more likely to engage in this behavior in the future. Each child will weigh the risk–benefit ratio of rewards to consequences differently. As documented by the fear avoidance model, avoidance is strongly associated with higher pain-related disability in this population. However, there is little research on the positive impact of engagement behaviors and how to promote these behaviors in youth with chronic pain. It is likely that engagement behaviors reduce pain intensity, anxiety, and depression and promote social interaction and normal functioning. However, engagement behaviors are not synonymous with positive outcomes and equally, avoidance behaviors may not be a proxy for disability. Indeed, in studies of adults with chronic pain, the engagement and persistence of activities has been linked to higher pain intensity [53]. Research investigating the consequences of goal pursuit is likely to be complex and involve dynamic processes that may change over the course of a child’s pain condition.

## 4. Future Directions

We present a new social-environmental model of goal pursuit behavior in order to advance research on understanding children’s and parent’s responses to chronic pain and potential strategies to promote engagement behaviors and return to normal functioning. In particular, we identified three areas in the model that are important to understand. First, research is needed to understand what inhibits and motivates important goals for youth with chronic pain, apart from the pain context itself. Child and parent factors outlined in the model are suggested as a starting point. For example, relevant research questions are whether parent protectiveness or expectations influence the pursuit of salient goals; how much motivation is needed for a child to approach a goal instead of avoid it; and whether there are associations between self-efficacy, the child’s pain experience, and their goal pursuit behaviors. Further exploration of the interaction between child and parent factors will provide a more comprehensive understanding of children’s goal-driven behavior in the context of chronic pain. Further, more research is needed to determine whether approach and avoidance behaviors lead to negative or positive outcomes for this population. As discussed previously, engagement can lead to higher pain and disability in adults [53], but this has not been studied in youth. Similarly, identifying whether goals are realistic or have been “given up” by youth may also be important within the context of approaching and avoiding. Goals can be both short- and long-term, and we do not differentiate between these in our model. Frequent everyday activity may be both a short-term goal to stay active, but also a long-term goal to have good physical conditioning. However, youth may not always recognize long-term goals, or that immediate actions may lead to the attainment of long-term goals.

Second, research investigating goal conflict is important in order to understand the tipping balance between engagement and avoidance of goals when experiencing pain. There is emerging research in adults using experimental methods of pain and altering goals using monetary rewards to investigate this question [38,54]. Within pediatrics, vignettes have been used to understand goal conflict and approach–avoidance behaviors in healthy youth [41]. However, little research has been conducted with youth with chronic pain on goal conflict to understand whether youth with chronic pain have frequent goal conflict and how they attempt to resolve it. Studies might employ experimental methodologies or ecological momentary assessment to investigate situations where high pain intensity conflicts with highly salient goals and to determine which individual factors predict engagement or avoidance.

Third, psychological therapies for youth with chronic pain could be developed or refined to reduce goal frustration and conflict and to aid problem solving when a goal cannot be engaged. Factors outlined in the Goal Pursuit model could be targeted. For example, treatments could target increasing children’s self-efficacy and motivation, which we predict will increase the likelihood of goal engagement. Strategies could also employ already established strategies to decrease pain-related anxiety and general anxiety [55], which may in turn reduce goal pursuit barriers. Parent factors are also crucial to address, and treatments are increasingly including parent interventions to improve communication, coping skills, and parenting behaviors [56]. Parent treatments have recently been piloted for this population and have shown benefit for reducing parent distress as well as positive but preliminary “downward” effects for improving children’s emotional functioning [57]. So far, the active ingredients that lead to change within these psychological treatments are unknown; a more frequent use of conceptual models such as the one proposed here to design and evaluate treatments could enhance understanding and inform modifications to enhance the effectiveness of these therapies.

Another avenue of investigation is goal setting within the context of psychological or other pain management treatments. Within cognitive behavioral treatments, therapists may set goals directly with children and parents and may encourage parents to appropriately support the chosen goal and even provide a reward system for reaching targeted goals. As discussed, parents may be particularly important in supporting youth in reaching their goals by providing encouragement and setting behavioral expectations consistent with engagement. There is some evidence to suggest that treatment goals are more effective if mutually agreed between parent and teen [29]. However, further research is needed to explore the content of goals as well as their salience in children and their parents entering treatment to understand their potential influence on treatment engagement and outcomes.

Finally, our model is not exhaustive of all factors that could be investigated when considering goal pursuit. We have specifically targeted psychosocial factors in our conceptualization of goal pursuit, but there are other neurobiological factors (e.g., sex, genetics, and cognition) that may also be important to consider.

## 5. Conclusions

In summary, we propose a new model of goal pursuit in pediatric chronic pain to guide research and clinical assessment of children’s responses to chronic pain. This model offers a broader perspective on youth engagement versus avoidance of activities among youth who are actively seeking goals despite their pain. A more complete understanding of child and parent factors that influence goal pursuit and goal salience is needed to further advance and motivate treatment development to enhance the effectiveness of psychological treatments for youth with chronic pain.
